# Cancer Burden Among Arab-World Females in 2020: Working Toward Improving Outcomes

**DOI:** 10.1200/GO.21.00415

**Published:** 2022-03-08

**Authors:** Hala Mahdi, Layth Mula-Hussain, Zhian S. Ramzi, Marwan Tolba, Omar Abdel-Rahman, Ibrahim Abu-Gheida, Ola Khorshid, Sana Al Sukhun, Nadeem P. Siddiqi, Zahid Al Mandhari, Maysa Al Hussaini

**Affiliations:** ^1^ Faculty of Health Sciences, McMaster University, Ontario, Canada; ^2^ Sultan Qaboos Comprehensive Cancer Care and Research Centre, Al Khoud, Muscat, Oman; ^3^ College of Nursing, University of Sulaimani, Sulaimani, Kurdistan, Iraq; ^4^ McGill University, Montreal, Quebec, Canada; ^5^ Cross Cancer Institute, University of Alberta, Edmonton, Alberta, Canada; ^6^ Burjeel Cancer Institute, Burjeel Medical City, Abu Dhabi, United Arab Emirates; ^7^ National Cancer Institute, Cairo University, Cairo, Egypt; ^8^ Al Hyatt Oncology Practice, Amman, Jordan; ^9^ King Hussein Cancer Center, Amman, Jordan

## Abstract

**PURPOSE:**

Cancer is the leading cause of morbidity and mortality worldwide. This work presents the Arab-world females' cancers (AFCs) statistics in 2020, compared with the 2018 AFCs statistics, the Arab-world male cancers statistics, and the world females' cancers (WFCs) statistics in 2020. This can help set the stage for a better policy for cancer control programs and improve outcomes.

**MATERIALS AND METHODS:**

A descriptive review of the 2020 Global Cancer Observatory concerning AFCs was performed. Data on various cancers were compiled and compared among the countries in the region and WFCs.

**RESULTS:**

A total estimate of 244,317 new cases and 132,249 deaths is reported in AFCs; representing 2.65% and 2.99% of WFCs, respectively, with an average crude incidence/mortality ratio of 116.2 (/100,000 population)/62.9 (/100,000 population) and an age-standardized incidence/mortality ratio of 137.7(/100,000 population)/77.2(/100,000 population) compared with 238.8(/100,000 population)/114.6(/100,000 population) and 186(/100,000 population)/84.2(/100,000 population) of WFCs, respectively. Five-year prevalent cases were 585,295; 2.28% of WFCs. In comparison to males, females accounted for 47.8% of the whole population, 52.9% in incidence, 46.9% in mortality, and 56.9% in the prevalence of patients with cancer. Mortality-to-incidence ratio (MIR) was 0.54 (range 0.39-0.62 in Arab countries, compared with 0.48 globally), and it ranged from 0.14 to 0.97 in the 30 AFC types. Breast cancer was the most common cancer in incidence and mortality, with an MIR of 0.39.

**CONCLUSION:**

The 2020 descriptive analysis of the females' cancers in the Arab world revealed a relatively high MIR compared with females' cancers worldwide; a lower MIR compared with the males; and comparable MIR to 2018 one. We call for more in-depth studies to determine the causes of these differences that might translate into actionable interventions and better outcomes.

## INTRODUCTION

Cancer is one of the top three causes of death worldwide, with one in six women developing cancer during their lifetime and one in 11 women dying from the disease.^1^ In 2020, it was estimated that globally, there were more than 50 million patients with cancer, with around 20 million incident cases and 10 million cancer deaths.^2^ The burden of cancer incidence and mortality is expected to double by 2040.^3^

**CONTEXT**

**Key Objective**
Cancer is the leading cause of morbidity and mortality worldwide. This work addresses the Arab-world females' cancers in 2020, not linked to sex only, compared with different cohorts, to help improve cancer control outcomes. It is a descriptive review from the latest Global Cancer Observatory.
**Knowledge Generated**
It showed an estimate of 244,317 new cases and 132,249 deaths, representing 2.65% and 2.99% of the world's peers, respectively. Compared with Arab-world males, females accounted for 47.8% of the population, 52.9% in incidence, 46.9% in mortality, and 56.9% in prevalence. The mean mortality-to-incidence ratio was 0.54 in Arab-world females, compared with 0.48 in world's females and 0.68 in the Arab-world males.
**Relevance**
We call for more in-depth studies to determine the causes of these differences that might translate into actionable interventions and better cancer control policies and management outcomes in Arab females.

Females globally constitute approximately 49.6% of the population (3,866,220,226 out of the total 7,794,798,844 in 2020). According to the 2020 Global Cancer Observatory (GCO) registry, females constituted 47.8% of the total 19,292,789 global incident cases, 44.5% of the total 9,958,133 deaths, and 50.9% of the total 50,550,287 5-year prevalent cases.^2^ That combined leads to the conclusion that, globally, cancer in females may be slightly more curable with better outcomes than males, a fact that was previously reported by other scholars.^4,5^

Although the cancer burden is an increasingly growing global concern, approximately 70% of cancer deaths occur in low- and middle-income countries. This is due to a host of factors, including the late diagnosis of the disease and inaccessibility to novel treatments.^6^ Although comprehensive cancer treatment is available in only 15% of low-income countries (LICs), it is available in more than 90% of high-income countries (HICs).^7^

The League of Arab States comprises 22 countries across the Middle East and North Africa region, with females representing 47.8% of the total population of approximately 436 million. Although these countries share historical, cultural, and geopolitical characteristics, the economy, human resources, and development vary widely. The countries in the Middle East and North Africa region are classified into three categories according to their gross national income: LICs, including Comoros, Djibouti, Mauritania, Yemen, and Somalia; middle-income countries, including Algeria, Egypt, Iraq, Jordan, Lebanon, Libya, Morocco, Palestine, Sudan, Syria, and Tunisia; and HICs, including Bahrain, Kuwait, Oman, Qatar, the Kingdom of Saudi Arabia, and the United Arab Emirates. Cancer incidence rates are predicted to continue rising in these countries, indicating that its future burden in the Arab world will increase as well, mirroring the global trend.^8^

Despite the apparent disparities in the global cancer burden in women,^9–11^ the literature on females' cancers in Arab countries (abbreviated as Arab-world females' cancers [AFCs]) is scarce. Before this work, we published data on the 2018 cancer statistics of females in the Arab world.^12^ In this work, we aim to describe and analyze the top 30 types of AFCs in 2020, whether sex-related or non–sex-related. We have also explored comparisons between the 2020 AFCs and 2018 AFCs, 2020 world females' cancers (WFCs), and the Arab-world males' cancers in 2020.

## MATERIALS AND METHODS

The 2020 GCO, an international cancer database, was used as the primary source of the findings of this work.^2^ A literature search was conducted on November 28, 2021, via PubMed medical search engine using the words: ((“Female”[Mesh]) AND “Arab World”[Mesh]) AND “Neoplasms”[Mesh], with 11 results, four of which interject with parts of this comprehensive work about the AFCs, mostly addressing sex-related cancers. These manuscripts were as follows: one is general, describing noncommunicable diseases in the Arab world,^13^ one describing ovarian cancer,^14^ and the last two describing breast cancer.^15,16^ Google Scholar was also searched; the results were mostly country- and cancer-specific and did not comprehensively cover the AFC spectrum. Therefore, we compiled the incidence, mortality, and prevalence rates from the GCO, and we calculated the mortality-to-incidence ratio (MIR) for each country and cancer type accordingly. Using the GCO, we also compared the 2020 statistics with those from 2018. In addition, we calculated and compared the female-to-male ratios for the 2020 population, cancer incidence, mortality, prevalence, and MIR in the Arabic countries and globally.

Simple descriptive statistics were used to calculate the frequencies and percentages. The crude incidence rate, age-standardized incidence rate (ASIR), the crude mortality rate, and the age-standardized mortality rate (ASMR) were calculated by the GCO. This was possible by entering the names of the Arab-world countries to extract these rates in the females' cancers. The Microsoft Excel for Mac (version 16.55) was used for the MIR calculations and to calculate other related measures and findings in this project. MIRs for the cancers, countries, and world groups were calculated by dividing the mortality cases over the incident cases.

## RESULTS

In 2020, the population of the countries of the Arab world was estimated at 436,378,884, which accounts for 5.6% of the total global population, out of which 47.8% were females (24.8-50.4). There were 463,675 new cancer cases estimated to be in Arab countries, corresponding to 2.4% of the global incidence. Females' cancers accounted for 52.9% of that number, compared with 47.8% worldwide. The ASIR for Arab females was 137.7/100,000, compared with the 186/100,000 population rate of WFCs. The total number of deaths was estimated to be 281,656, accounting for 2.8% of the global deaths, and females' deaths accounted for 46.9% of that total, compared with 44.5% worldwide. The ASMR was 77.2/100,000 population, compared with a rate of 84.2/100,000 population in WFCs. The 5-year prevalent cases were estimated to be 1,027,939, constituting 2% of the global 5-year prevalent cases, and Arab females constituted 56.9% of the Arab countries' survivors, compared with world females constituting 50.9% of the global survivors. The average MIR was 0.54, compared with 0.48 globally, and it ranged from 0.33, the most favorable, to 0.69, the least favorable. More details on these statistics from 2020 can be found in Table 1.

**TABLE 1:**
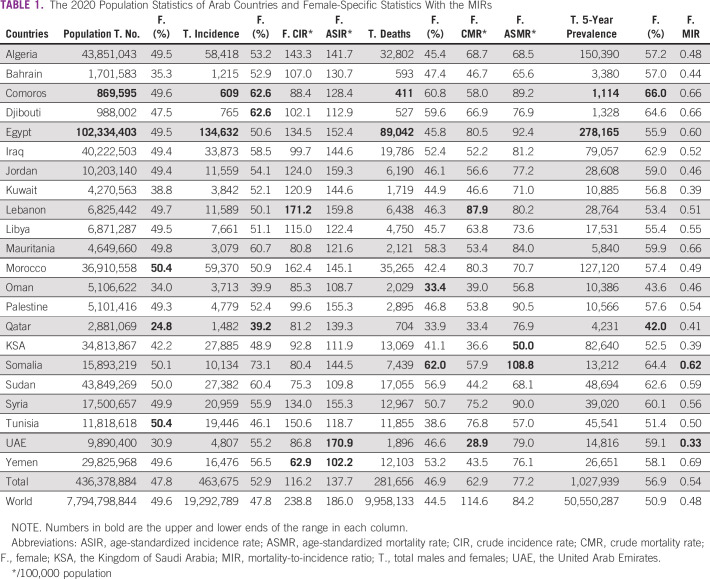
The 2020 Population Statistics of Arab Countries and Female-Specific Statistics With the MIRs

Compared with 2018, the Arab countries' population increased by 3% in 2020, from 422,717,439 to 436,378,884, and the females' proportion increased from 45.7% to 47.8%. However, the females' proportion of the incident cases decreased from 54% to 52.9%, their proportion of total deaths decreased from 48% to 46.9%, and their proportion of 5-year prevalent cases decreased from 58.4% to 56.9%. Interestingly, females' ASIR increased from 134.5/100,000 to 137.7/100,000 population, and the ASMR also increased from 75.4/100,000 to 77.2/100,000. Finally, females' MIR remained stable at 0.54. Tables 1 and 2 include the details on 2020 (Table 1) and 2018 (Table 2) statistics.

**TABLE 2:**
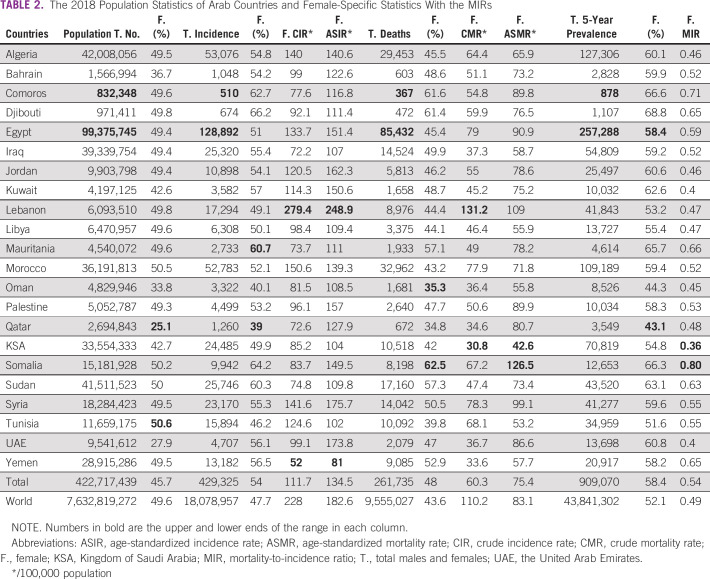
The 2018 Population Statistics of Arab Countries and Female-Specific Statistics With the MIRs

The 30 cancers included in the AFC category in 2020 are detailed in Table 3 (Table 4 shows the comparable statistics in 2018). Breast cancer was the top cancer in incidence, crude incidence rate, ASIR, deaths, crude mortality rate, and ASMR. By contrast, Kaposi sarcoma was the lowest in those measures. The lowest MIR (0.14) was observed for thyroid cancer, whereas the highest was for pancreatic cancer (0.97). Compared with 2018, the 30 cancer types in Arab females in 2020 increased by 6.9% (227,494 in 2018 to 244,317 in 2020), albeit the population of Arab-world females also increased by 7.4% during the same period, which means that the growth in incidence parallel that of the population. This also applies to the 7.1% increase in Arab WFC deaths (122,903 in 2018 to 132,249 in 2020), which, despite this increase, is still very similar to the population growth rate. The MIR, as aforementioned, remained stable at 0.54.

**TABLE 3:**
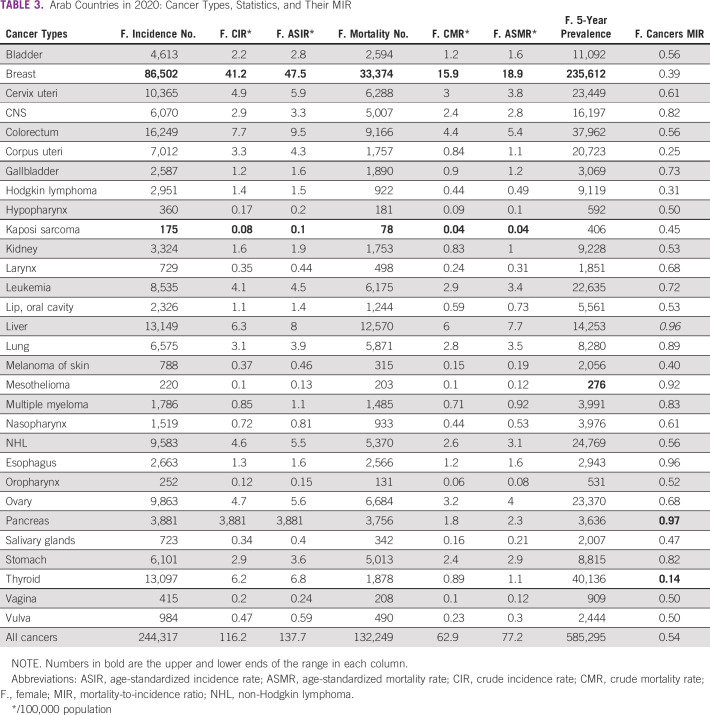
Arab Countries in 2020: Cancer Types, Statistics, and Their MIR

**TABLE 4:**
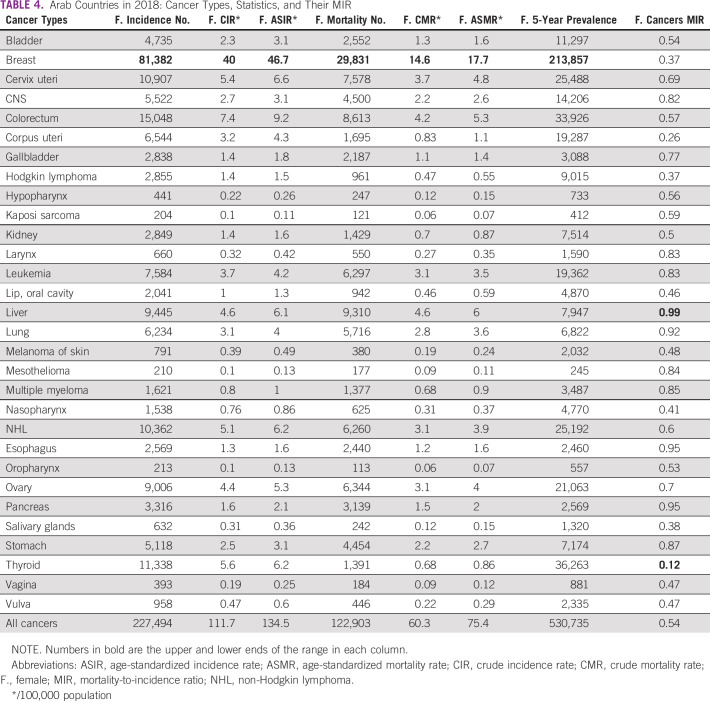
Arab Countries in 2018: Cancer Types, Statistics, and Their MIR

Upon comparing the top 10 cancers in 2020 between AFCs and WFCs, we observed that nine cancers are common in terms of incidence (breast, colorectum, liver, thyroid, cervix uteri, corpus uteri, ovary, lung, and non-Hodgkin lymphoma), with variation in the order of ranking. Leukemia was among the list for AFCs, whereas stomach cancer was among the list for WFCs. The mortality list revealed that eight cancers were common among WFCs and AFCs (breast, liver, colorectum, ovary, cervix uteri, leukemia, lung, and stomach) with variation in the rank. CNS cancer and non-Hodgkin lymphoma were among the list in AFCs, whereas esophagus and pancreas cancers were among the list of WFCs. The complete list of the top 10 cancers in Arab-world females and world females for 2018 and 2020 can be found in Table 5.

**TABLE 5:**
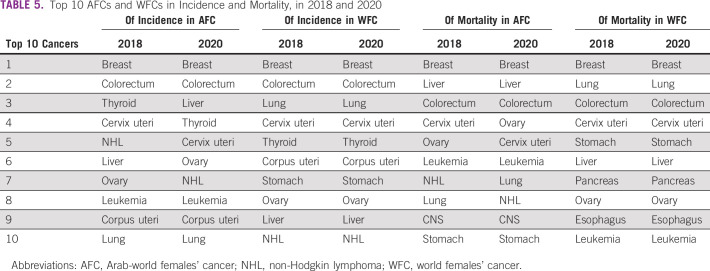
Top 10 AFCs and WFCs in Incidence and Mortality, in 2018 and 2020

Comparing the AFCs statistics with the world identified groups of human development index (HDI, which is classified into four categories, as follows: countries with very high HDI, high HDI, medium HDI, and low HDI) and income level (which are classified into another four categories, as follows: high-income country [HIC]; upper middle-income country [UMIC]; and lower middle-income country [LMIC]; and low-income country [LIC]). Table 6 shows the incident cases, ASIR, deaths, ASMR, and MIR for AFC in 2018 and 2020, compared with the parallel measures in WFC in general, and after that to those measures in very high HDI, high HDI, medium HDI, and low HDI, and the measures in HIC, UMIC, LMIC, and LIC. The details can be viewed in Table 6.

**TABLE 6:**
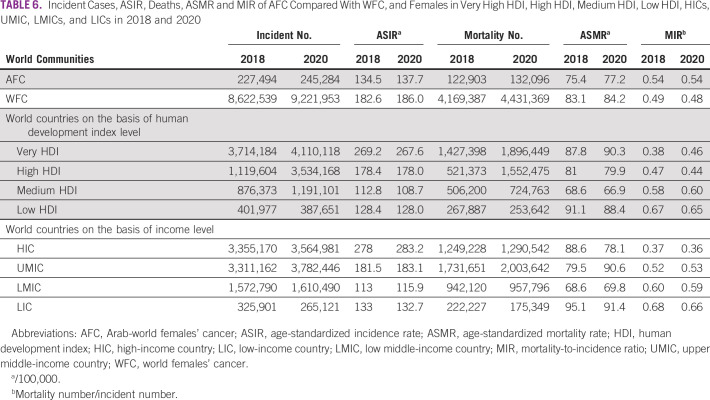
Incident Cases, ASIR, Deaths, ASMR and MIR of AFC Compared With WFC, and Females in Very High HDI, High HDI, Medium HDI, Low HDI, HICs, UMIC, LMICs, and LICs in 2018 and 2020

## DISCUSSION

In 2020, females constituted 47.8% of the Arab-world population, and the female cancer incidence, mortality, and 5-year prevalence constituted 52.9%, 46.9%, and 56.9% of the Arab world, respectively. The female MIR average was 0.54, ranging from 0.33 to 0.69, compared with a global average of 0.48. Breast cancer ranked the first in incidence and deaths, whereas Kaposi sarcoma ranked the last for both parameters. Although the highest MIR was seen with pancreatic cancer (0.97), thyroid cancer had the lowest MIR (0.14). After breast cancer, and in descending order, cancers of the colorectum, lung, cervix uteri, and thyroid followed in incidence, whereas cancers of the liver, colorectum, ovary, and cervix uteri followed in mortality. Mesothelioma was the least prevalent cancer type.

This study comes in sequence, and as a follow-up study, after the manuscript on Arab-world females' cancers on the basis of 2018 statistics,^12^ the poster that was presented during the 9th Arab Health Summit on the 2020 Arab-world females' cancers,^17^ and the 2020 Arab-world males' cancers manuscript, currently submitted. Comparing the MIR between males and females in the Arab world and globally, we noticed that MIR is lower in females than males in both cohorts: 0.54 in 2018 and 2020 for the females in the Arab world, and 0.49 and 0.48, respectively, globally. This is compared with males in the Arab-world MIR: 0.69 in 2018 and 0.68 in 2020 compared with 0.57 in 2018 and 0.55 in 2020, globally. This might indicate that female cancers might be less aggressive and more curable than male cancers, which was reported previously,^4,5^ although they are occurring at a higher incidence rate. This is probably driven by consistently higher incidence and prevalence of breast cancer.

Moreover, Arab females account for 47.8% of the population; they account for 52.9% of incident cases, 46.9% of deaths, and 56.9% of 5-year prevalent cases. When comparing this to the global females' data, females account for 49.6% of the total population, 47.8% of incident cases, 44.5% of deaths, and 50.9% of prevalent cases. Despite that, these numbers may show that Arab-world females appear to have higher rates of cancer and cancer mortality than females globally and more unfavorable outcomes as seen by the differences in MIR (0.54 for AFCs and 0.48 for WFCs). Still, one must be cautious as, without statistically significant associations, the authors do not think they can draw conclusions on the basis of numbers only.

It is important to note that the gap between the female Arab-world and global MIR (0.06) and the outcome might point to the probable health care access and care inequity among Arab females compared with females globally. The lower MIR in females compared with males both in the Arab world and globally could be attributed to the relatively good outcome associated with the most common cancer in the females, breast cancer, related to media-focused screening campaigns, early detection, and modern therapies led to lower mortality in females. Additionally, health care access disparities, the high smoking rates, and associated malignancies in males in the Arab world compared with females,^18^ and the reluctance to early seeking medical attention in Arab males, might account for this difference.^19^ There is growing body of sex-specific studies that highlight a trend of males' delayed help seeking when they become ill.^20^ However, cancer incidence and outcome represent a complex interaction between lifestyle, inherent differences in tumor biology, genetic, socioeconomic, and environmental factors. All may interplay to contribute to this difference in MIR.

Aside from differences between the males' and females' statistics, there were several prominent differences between the data within Arab countries and worldwide data. The ASIR and ASMR for Arab-world females versus global females were 137.7/100,000 versus 186/100,000 and 77.2/100,000 versus 84.2/100,000, respectively. However, these lower incidence and death rates might contradict MIR in the two cohorts, as the average value for Arab countries was 0.54, whereas the global average was 0.48. Again, these regional and worldwide data trends might indicate that although females in Arab countries have lower incidence and mortality rates than females globally, they generally have less favorable outcomes, as seen from the higher MIR. This observation indicates that there is room to improve comprehensive cancer care, starting from early detection and education, better access to modern therapeutics and interventions, and supportive and palliative care.^21^

Comparing data sets from 2018 and 2020, it is important to note that although the proportion of Arab-world females increased from 45.7% to 47.8%, their proportion with regards to incidence, mortality, and 5-year prevalent cases all decreased from 54% to 52.9%, 48% to 46.9%, and 58.4% to 56.9%, respectively. Although the decrease of incidence and mortality are positive trends, the reduction of the 5-year prevalent cases indicates a lowered long-term survival rate. Other notable changes are that the female ASIR increased from 134.5/100,000 in 2018 to 137.7/100,000 in 2020, and the female ASMR also increased from 75.4/100,000 in 2018 to 77.2/100,000 in 2020. Finally, as the MIR remained stable from 2018 to 2020 at 0.54, this indicates relative stability in the outcome of female cancers in the Arab world during the 2 years.

Furthermore, a relative improvement in the most aggressive cancers' MIR was observed between 2018 and 2020, including liver (0.99-0.96), lung (0.92-0.89), stomach (0.87-0.82), and leukemia (0.83-0.72), excluding the esophagus (0.95-0.96), which increased, and the pancreas, which remained stable (0.95). This modest improvement, especially for liver cancer, which previously had the most unfavorable MIR, might reflect the advances in cancer screening, comprehensive multidisciplinary and evidence-based management guidelines,^22^ including improved surgical approaches, precision radiotherapy techniques (such as stereotactic ablative and image-guided radiosurgery and radiotherapy),^23^ and adoption of new targeted and immunotherapeutic options.

Although breast cancer is the leading cancer in terms of incidence and mortality in AFCs, its MIR is relatively low (0.39), and its 5-year prevalent cases are also relatively high (234,612). This indicates that although it is pervasive, which occurs at high rates among the Arab-world females (35% of the females' cancer cases are breast cancers), it appears to be less aggressive and has a relatively good outcome, probably attributable to early detection (at least, in some of the countries) and effective therapies in general. However, as previously mentioned, the MIR for breast cancer increased from 0.37 in 2018 to 0.39 in 2020, indicating a possible negative trend. This should be further investigated in the coming years to observe whether the trend continues. Another trend is that the MIR for leukemia has significantly decreased from 2018 (0.83) to 2020 (0.72), likely because of its better management globally. By contrast, although lung cancer ranks tenth for incidence in AFCs, it is third in WFCs, a difference attributed to the relatively lesser use of tobacco among the Arab world, primarily because of cultural and societal norms.

Additionally, there were disparities in MIR changes from 2018 to 2020 for specific Arab countries. In Algeria, Lebanon, Libya, the Kingdom of Saudi Arabia, and Yemen, the MIR unfavorably increased from 0.46, 0.47, 0.47, 0.36, and 0.65, to 0.48, 0.51, 0.55, 0.39, and 0.69, respectively. By contrast, the MIR favorably decreased in Bahrain, Comoros, Morocco, Qatar, Somalia, Sudan, Tunisia, and the United Arab Emirates from 0.52, 0.71, 0.52, 0.48, 0.80, 0.63, 0.55, and 0.40, to 0.44, 0.66, 0.49, 0.41, 0.62, 0.59, 0.50, and 0.33, respectively. The unfavorable changes are likely because of disparities in health care access and the quality of services between the Arab countries, income level and gross domestic product, and war and geopolitical status in different Arab nations, namely Lebanon, Libya, Algeria, and Yemen.^24^ However, the significant number of countries with improved female MIRs shows a promising trend for the future, indicating improvements in screening, detection, treatments, and overall outcomes.

Notably, in AFCs, liver cancer ranks third in incidence (with 13,149 new cases, after breast and colorectal cancers), and it ranks second in deaths (with 12,570 deaths, after breast), compared with the ninth rank among WFCs for incidence and sixth in fatalities. Although liver cancer is not prevalent in most Arab countries, this is probably attributed to the high burden and prevalence of liver cancer in the Arab country with the largest population, Egypt, where the population accounts for approximately a fourth of the Arab-world population at over 102 million. Among the total liver cases in AFCs in 2020, Egypt constituted 74% of the incidence and deaths of liver AFCs (9,750 incident cases and 9,312 deaths). Liver cancer in Egypt ranked first in the total incidence in 2020 with 27,895 cases (20.7% of the total cancer cases for both males and females), and second in females, just after breast cancer, which was estimated to be 22,038 cases. Egypt has been suffering from a high burden of hepatitis, which is the most significant risk factor in developing liver cancer.^25^ Since Egypt has the highest prevalence of hepatitis C virus infection worldwide, the health authorities in 2018 started a successful national screening and treatment campaign inclusive of about 50 million of the population.^26^ Hopefully, continuing in this direction in the future will decrease the incidence and improve the outcome of this aggressive cancer.

It is also interesting to compare the top 10 cancers in AFCs between 2018 and 2020, as there are some apparent similarities and differences. First, in terms of incidence and mortality, the top two cancers remained the same (breast and colorectum for incidence and breast and liver for mortality). Although the order of the remaining top cancers varies slightly between 2018 and 2020, it is significant to note that the same 10 cancers were present in the incidence and mortality lists. Even in terms of the order, the changes across the 2 years were typically only one or two places apart; for example, cervix uteri cancer ranked fourth in incidence in 2018 but fifth in 2020.

Although the MIR in individual countries in the Arab world may be similar to the measures in their peer countries of similar HDI and income level, the average of MIR in AFCs (0.54) can fit in the range of MIR between high to medium HDI (0.44-0.60) and in the range of UMIC to LMIC (0.53-0.59) as per the 2020 MIR measures in Table 6. This highlights the importance of the external factors, such as the development and income, and their impact on the MIR and cancer burden.^24^

The trends of AFCs seen in these data prove the need for extensive analysis and call for more action. Some literature supports the prediction that the burden of cancer in the Middle East will duplicate over the coming decade.^27^ Although many Arab-world countries have national cancer control policies (80%), only 45% of the programs are operational and that the amount of literature and research conducted in this field remains insufficient, particularly with regards to studies on preventative policies.^28^ It is essential to emphasize the need for more oncology research in the Arab world,^29^ and the right to health, and that the patients with cancer need more attention to decrease the burden and improve the outcomes.^30^

The strengths of this work include the use of the most recent and up-to-date estimates of global and Arab-world cancer statistics, the authors' multinational collaboration, and being the second collective review concerning the spectrum AFCs. In addition, comparison to cancer rates in females globally and males in the Arab world adds to its value. This study also uniquely compares the AFC statistics from 2018 and 2020, showing how specific data changed, hypothesizing potential causes for the change. Importantly, our study can pave the road for more in-depth follow-up studies and analyses. However, we cannot overlook the limitations of this work, as this is a descriptive study on the basis of an estimate from retrospectively collected data from a comprehensive global database. Furthermore, the lack of reliable resources, data reporting, and documentation in many Arab-world countries might hinder the accuracy of many of the used statistics and the perfection of this initiative.

In conclusion, the 2020 descriptive analysis of the females' cancers in the Arab world revealed a relatively high MIR compared with females' cancers worldwide; a lower MIR compared with the males; and comparable MIR to 2018 one. We call for more in-depth studies to determine the causes of these differences that might translate into actionable interventions and better outcomes.
